# Supplementary material for: Short-Term Impact of Recycling-Derived Fertilizers on Their P Supply for Perennial Ryegrass (Lolium perenne)
Source: Plants (Basel). 2023 Jul 25;12(15):2762. doi: 10.3390/plants12152762 (PMC10420916; doi:10.3390/plants12152762)
Supplement: Supplementary file 1 [file plants-12-02762-s001.zip › plants-2515197-supplementary.pdf]

plants

MDPI

Short Term Impact of Recycling-Derived Fertilizers on their P Supply for Perennial Ryegrass (*Lolium perenne*)

Lea Deinert[1], Israel Ikoyi[1,2], Bastian Egeter[3‡], Patrick Forrestal[4], Achim Schmalenberger[1*]

Supplementary information

S1 – MPN calculation details

This value must be multiplied with the dilution factor of the highest dilution, where all wells were positive for microbial growth, then the dilution factor for the addition to the well (50 µL mL^{-1}), the dilution factor for the initial soil sample amount (in grams) applied in saline solution and the final result was expressed as MPN g soil^{-1}, always in conjunction with the P source that had been applied to the minimal medium.

$$\frac{MPN}{g\ soil} = \frac{MPN\ value\ \cdot Dilution\ Factor\ \cdot 50\ \frac{\mu L}{mL}}{Soil\ Weight\ (g\ soil)}$$

S2 – Calculating CFU from TCP experiment

The cultivation of soil microbes on the solid medium supplemented with tri-calcium phosphate was analysed by counting the colonies which formed a halo around them, a clearing zone indicating the solubilization of the TCP in the agar. The count was then multiplied with the dilution factor of the inoculation, corrected for the volume from 100 µL to 1 mL and adjusted for the soil weight used for the microbial extraction:

$$\frac{CFU}{g\ soil} = \frac{Mean\ Colony\ Count\ \cdot Dilution\ Factor\ \cdot 10\frac{\mu L}{mL}}{Soil\ Weight\ (g\ soil)}$$

S3 – Potential acid and alkaline phosphomonoesterase activity measurements

First, 1 g of soil (sieved, 2 mm mesh size) was mixed with 0.2 mL toluene, 4 mL modified universal buffer (either at pH 6.5 for acid phosphatase, or at pH 11 for alkaline phosphatase) and 1 mL 0.05 M p-nitrophenyl phosphate (pNP) as substrate for the hydrolytic reaction and then incubated in a water bath at 37°C for an hour. After the incubation had finished, 1 mL 0.5 M calcium chloride and 4 mL 0.5 M sodium hydroxide were added, and the solution was mixed to stop the enzyme reaction. Controls were prepared similarly as a composite sample for each treatment, however, the p-nitrophenyl phosphate solution was added after the enzymatic reaction had been stopped. This was done to correct for background signal originating from other sources than p-nitrophenol release in the sample. The soil suspension was then filtered through Grade 113 Cellulose qualitative filter paper (Fisherbrand, Fisher Scientific, UK) and the resulting p-nitrophenol was measured spectrophotometrically (M501 Single Beam Scanning UV/Visible Spectrophotometer, Camspec, UK) at 420 nm. The final p-nitrophenol concentration obtained in 1 g of soil after 1 h of substrate incubation was calculated via a standard curve by measuring the absorption for 0 (blank), 10, 20, 30, 40 and 50 µg p-nitrophenol and the result was stated in µg g soil^{-1} h^{-1}.

S4 – Morgan's P measurement

5 mL of dried soil were mixed with 25 mL Morgan's extractant solution (pH 4.8)on a horizontal shaker (GFL 3018, Burgwedel, Germany) for 30 min at 180 rpm. Then the solution was filtered using Grade 113 Cellulose qualitative filter paper (Fisherbrand, Fisher Scientific, UK). 1 mL of eluate was incubated with 4 mL colour development solution for 30 min, then the absorption of the antimony-phospho-molybdate complex was measured at 882 nm using a spectrophotometer (M501 Single Beam Scanning UV/Visible Spectrophotometer, Camspec, UK). The orthophosphate concentration was calculated via a standard curve prepared with Morgan's extractant and a 50 mg P L^{-1} potassium dihydrogen phosphate solution to obtain standards with a concentration of 0 (blank), 0.2, 0.5, 1, 2, 3, 4, 5, 6, 7 and 8 mg P L^{-1}.

S5 - PCR-DGGE

The addition of a GC-clamp to the forward primer improves stability in fragment separation during DGGE. In detail, a 25 µL reaction contained 1x DreamTaq buffer (2 mM $MgCl_2$, Fisher Scientific, Waltham, MA, USA), 1 M betaine, 2 mM dNTP mix, 0.4 mM of each primer (341F-GC 5′-CGC CCG CCG CGC GCG GCG GGC GGG GCG GGG GCA CGG GGG GCC TAC GGG AGG CAG CAG-3′ and 518R 5′-ATT ACC GCG GCT GCT GG-3′), 0.5 U DreamTaq polymerase (Fisher Scientific, Waltham, MA, USA) and 0.5 µL DNA template. The PCR touchdown protocol was applied in a Mini Amp Plus thermal cycler (Applied Biosystems, Thermo Fisher Scientific, Waltham, MA, USA) as follows: an initial denaturation at 95°C for 5 min, 20 cycles of touchdown annealing from 65 – 55°C (temperature lowered by 0.5°C every cycle), followed by 18 cycles of denaturation at 94°C for 45 s, annealing at 55°C for 45 s and elongation at 72°C for 60 s. Final extension was performed at 72°C for 10 min. A DGGE fingerprinting analysis was performed in a 10 % v/v polyacrylamide gel prepared in 1x TAE buffer in TV-400 DGGE system (Scie-plas, Cambridge, UK), with urea/formamide denaturing gradient of 35-65 % for the 16S rRNA analysis (for preparation details see chapter 7.5 in the appendix). Band patterns were stained with diluted SYBR™ Gold Nucleic Acid Stain (Invitrogen, Thermo Scientific, Leicestershire, UK) and imaged using a transilluminator (G:box, Syngene).

Supplementary figures

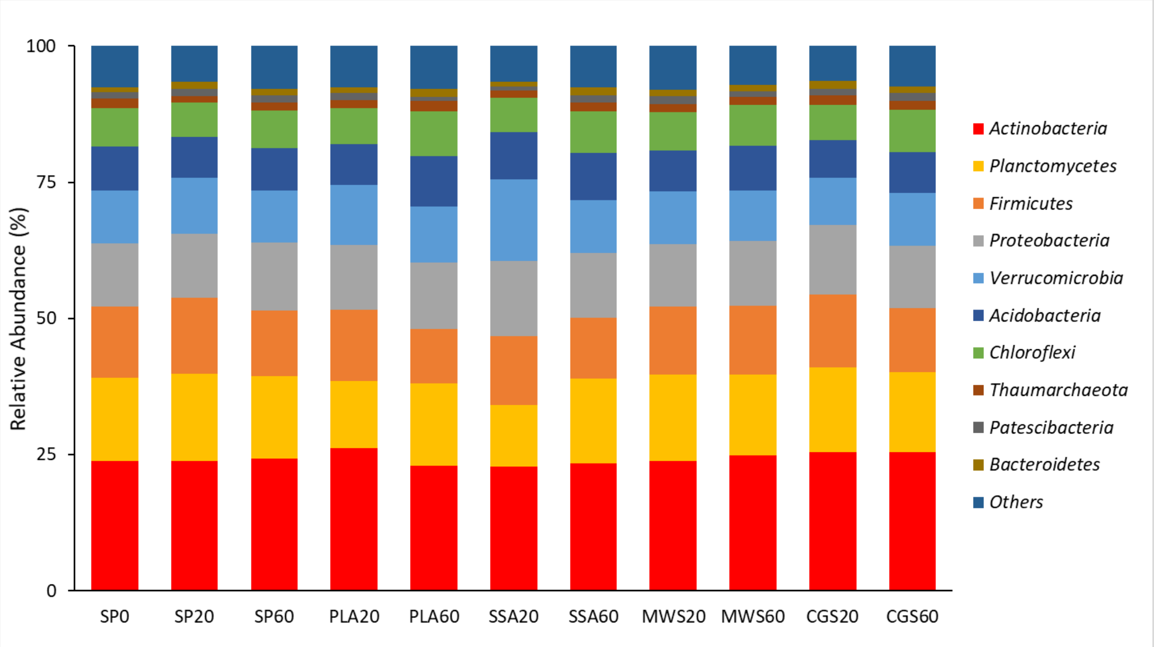

Figure S1. Mean relative abundance of the top 10 abundant phyla of the 16S rRNA amplicon sequencing, n=4.

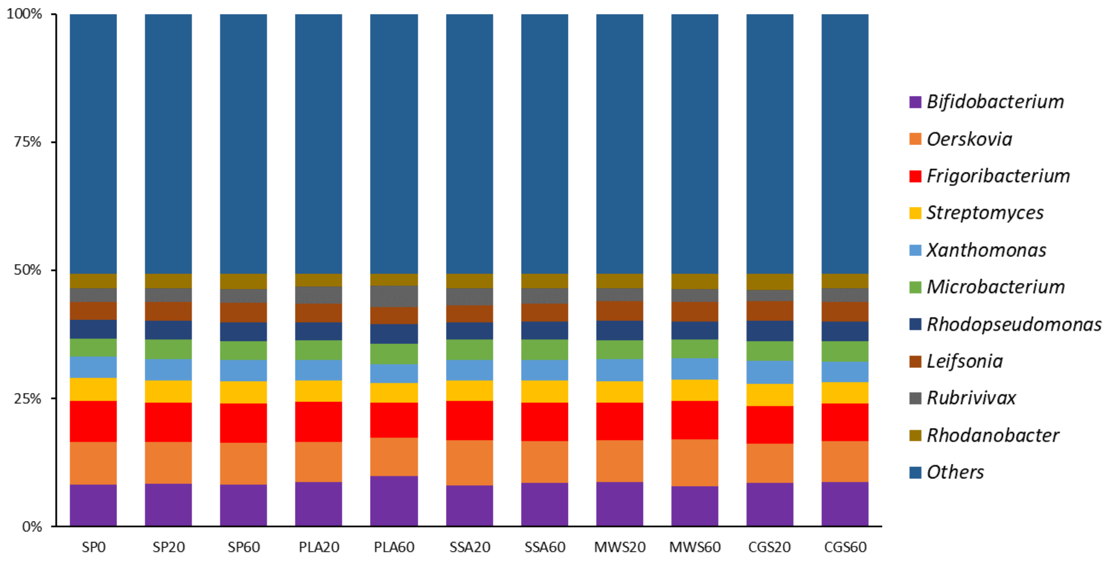

Figure S2. Mean relative abundance of the top 10 abundant genera of the phoD amplicon sequencing of the pot trial, all other genera collapsed in "others", n=4.

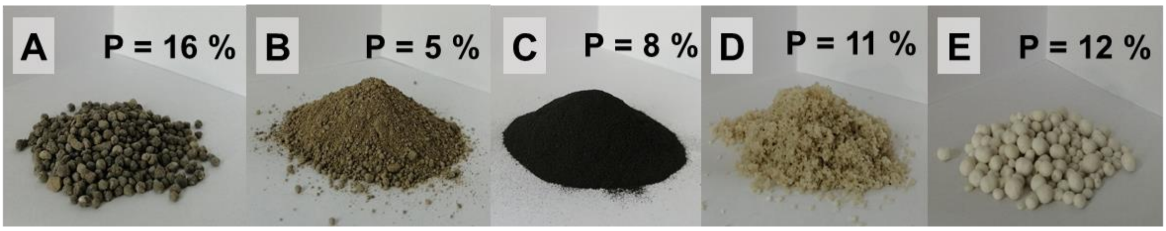

Figure S3. Images of P fertilizers applied in the pot trial with their P content given in percent: A) superphosphate (SP), followed by recycling-derived fertilizers B) poultry litter ash (PLA), C) sewage sludge ash (SSA), D) municipal wastewater struvite (MWS) and E) CrystalGreen® struvite (CGS).

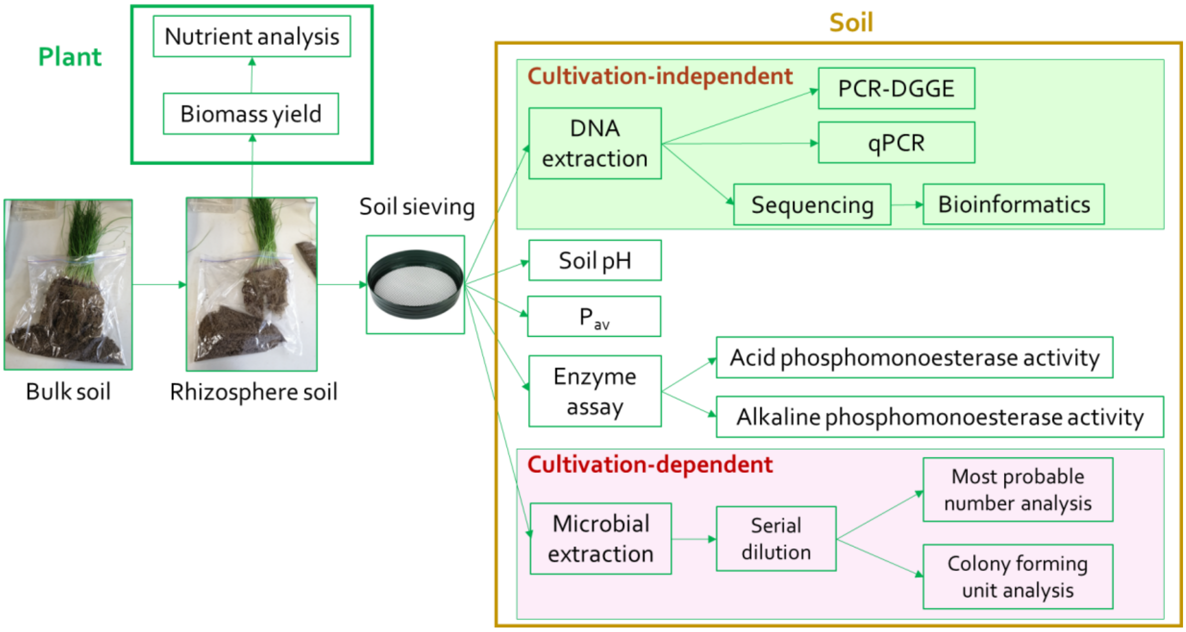

Figure S4. Experimental workflow of the pot trial post-harvest analyses.

Supplementary Tables

Table S1. Nutritional composition of the recycling-derived fertilizers applied in the experiment, the values for the RDFs PLA, SSA and MWS were evaluated by University of Ghent (ReNu2Farm report, WPT1 D3.1. Product Characterization) and the values for CGS were given on the company's website (https://crystalgreen.com/agriculture/).

	PLA	SSA	MWS	CGS
pH (KCl)	12.0	11.2	7.1	n.d.
Dry matter (%)	92.0	100.0	50.0	n.d.
Total Carbon (g kg^{-1} FW)	10.7	< 5.5	< 5.5	n.d.
Total Nitrogen (g kg^{-1} FW)	<0.9	<0.9	53.1	n.d.
NH_4-N (g kg^{-1} FW)	0.01	0.02	0.11	50
P (g kg^{-1} FW)	38.7	48.2	49.8	120
K (g kg^{-1} FW)	69.8	6.7	0.21	n.d.
S (g kg^{-1} FW)	19.2	39.9	0.04	n.d.
Na (g kg^{-1} FW)	9.1	104.6	0.03	n.d.
Ca (g kg^{-1} FW)	111.9	62.7	0.18	n.d.
Mg (g kg^{-1} FW)	26.5	8.9	44.3	100
Zn (mg kg^{-1} FW)	1592.9	1390.3	< 4.17	n.d.
Fe (mg kg^{-1} FW)	3708.8	31939.5	118.8	n.d.
Cu (mg kg^{-1} FW)	312.5	460.5	< 3.33	n.d.
Al (mg kg^{-1} FW)	4972.0	35541.6	< 6.67	n.d.

FW: fresh weight, n.d.: not determined.

Table 2. Nutrient application rates as recommended by Teagasc for grassland on which grazing livestock is kept, and the nutrient concentrations finally applied to the pots [52].

	Recommended Concentrations (kg ha^{-1})	Concentration Applied (kg ha^{-1})	Concentration (mg cm^{-2})	Mass per Pot (mg)
N	220	220	2.20	248.8
K	182	185	1.85	209.2
Ca	2 kg Ca / 1 kg N	440	4.40	497.6
S	20	20	0.20	22.6
Mg	30	30	0.12	13.7
$ZnSO_4$	5	5	0.05	5.7
$CuSO_4$	20	15	0.15	17.0
